# Low-light enhancement method with dual branch feature fusion and learnable regularized attention

**DOI:** 10.1007/s12200-024-00129-z

**Published:** 2024-08-14

**Authors:** Yixiang Sun, Mengyao Ni, Ming Zhao, Zhenyu Yang, Yuanlong Peng, Danhua Cao

**Affiliations:** 1 School of Optical and Electronic Information, Huazhong University of Science and Technology, Wuhan, 430074 China; 2 State Grid Information & Telecommunication Branch, Beijing, 100761 China

**Keywords:** Power inspection, Low-light enhancement, Feature fusion, Learnable regularized attention

## Abstract

Restricted by the lighting conditions, the images captured at night tend to suffer from color aberration, noise, and other unfavorable factors, making it difficult for subsequent vision-based applications. To solve this problem, we propose a two-stage size-controllable low-light enhancement method, named Dual Fusion Enhancement Net (DFEN). The whole algorithm is built on a double U-Net structure, implementing brightness adjustment and detail revision respectively. A dual branch feature fusion module is adopted to enhance its ability of feature extraction and aggregation. We also design a learnable regularized attention module to balance the enhancement effect on different regions. Besides, we introduce a cosine training strategy to smooth the transition of the training target from the brightness adjustment stage to the detail revision stage during the training process. The proposed DFEN is tested on several low-light datasets, and the experimental results demonstrate that the algorithm achieves superior enhancement results with the similar parameters. It is worth noting that the lightest DFEN model reaches 11 FPS for image size of 1224×1024 in an RTX 3090 GPU.

**Graphical Abstract:**

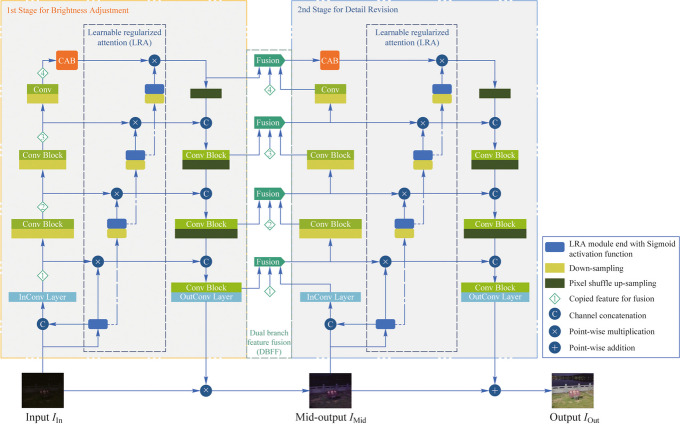

## Introduction

Power inspection is an essential component in ensuring the stable operation of the power grid. Currently, the mainstream inspection solution is to analyze the images captured by the monitoring equipment through manual or computer vision techniques [1–3]. However, images collected during night or bad weather scenes often suffer from insufficient lighting, resulting in reduced contrast and color distortion. These issues can affect both the subjective perception of the observer and the detection accuracy of subsequent computer vision systems. Therefore, how to improve the quality of images captured from low-light scenes has become an important area of research.

The development of computer vision technology has led to numerous studies on improving low-light images using enhancement algorithms. There are three main challenges when deploying low-light enhancement algorithms in practical engineering scenarios, which are enhancement performance, scene adaptability, and inference efficiency.

Existing low-light enhancement algorithms are less capable of addressing all three issues at the same time. The performance of traditional enhancement algorithms [4–10] depends heavily on the configuration of model parameters, which means they may be difficult to handle various scenarios. Although the lightweight neural network-based algorithms have high adaptive capacity [11–18], it is difficult to solve the color distortion and noise interference of low-light images due to the limitation of the model volume. Besides, the algorithms with outstanding enhancement performance and scene adaptability [19–27] have complex network structures, which make it hard to enhance large size images in edge device with weak computational capability rapidly.

In this paper, we propose a two-stage end-to-end low-light enhancement model called Dual Fusion Enhancement Network (DFEN) for efficient enhancement of low-light images of grid inspection scenes. Referring to the imaging steps of the digital camera [28], which first performs signal amplification and then executes the image signal processing, we decompose the low-light enhancement task into two stages of brightness enhancement and detail revision, which are implemented by two series-connected U-Nets sequentially. When the U-Net goes deeper, high-resolution details embedded in the low-level features tend to be partially missing after the scale transformation. Therefore, we introduce a dual branch feature fusion module that selectively reconstructs the same scale features of the two-stage network through the channel and spatial fusion branches. Furthermore, considering the significant differences of lighting conditions in various regions of the high dynamic range images, enhancing them according to a uniform standard may result in overexposure of bright regions. A learnable regularized attention module is introduced to extract the illumination attention map of low-light images, which can guide the model to adaptively enhance the low-light images.

The proposed method is validated on several datasets including our self-built Dark Grids dataset with multiple scenarios to verify the scene adaptation. The experiment results demonstrate that our algorithm can meet the demand for rapid enhancement of high-resolution images while achieving superior results on a variety of evaluation metrics compared to the state-of-the-arts. Our contributions are as follows:

1. We investigate a novel size-controllable low-light enhancement algorithm DFEN. It decomposes the low-light enhancement task into two stages of brightness enhancement and detail revision, allowing it to focus on different goals in each stage and achieves better enhancement results.
2. We adopt the dual branch feature fusion (DBFF) module to shorten the feature path of the algorithm and preserve the high-resolution information, which selectively aggregates features of the same scale in the spatial and channel dimensions. This module can effectively improve the texture detail preservation and color restoration ability of the DFEN model.
3. We design the learnable regularized attention (LRA) module to balance the enhancement effect of different regions, which can effectively suppress the overexposure in bright regions and further improve the scene adaptability of the algorithm.
4. For nighttime grid inspection scenes, we construct a paired low-light enhanced dataset containing multiple scenarios, called Dark Grids dataset, and the proposed DFEN outperforms the state-of-the-arts on several datasets including it.

## Related work

According to the algorithm principle, the low-light image enhancement methods can be divided into two categories. One is based on the Retinex theory, which decomposes low-light images into illumination and reflection images for separate processing. The other one enhances the low-light image directly without decomposition.

### Retinex-based low-light images enhancement methods

Retinex theory treats the observed image as the product of the illumination component $L$ and the reflection component $R$, i.e., $S = R \times L$, where $R$ is not affected by the non-uniformity of light. According to the Retinex theory, we can decompose the low-light image into illumination and reflection ones to process separately, then fuse them to obtain the enhanced image.

Jobson et al. respectively proposed Single-Scale Retinex (SSR) [4], Multi-Scale Retinex (MSR) [5], Multi-scale Retinex with Color Restoration (MSRCR) [6] in 1995, 1996, and 1997. SSR took the Gaussian surround function filtered image as the estimated illumination map. However, this method could not guarantee both the color fidelity of the image and the dynamic compression capability of the algorithm at the same time. To improve the robustness of SSR, MSR got the final illumination map by weighted averaging the multiple illumination maps obtained from different scales Gaussian kernels. MSRCR was proposed to solve the color-bias problem in SSR and MSR by introducing a color recovery factor C to adjust the ratio of RGB channels. LIME [7] proposed by Guo in 2017 extracted the maximum value of pixels in each color channel of the original image as the initial illumination map, and then optimized the illumination map by the Augmented Lagrangian Multiplier (ALM). In the same year, Ying et al. proposed a dual-exposure fusion algorithm BIMEF [8] to avoid excessive contrast and lightness over-enhancement. BIMEF fused the input image with the best exposure image generated by the camera response model according to the image fusion weight matrix to obtain the enhanced image. CRM [9] proposed by Ying et al. in 2017 used the camera response model to adjust each pixel to the desired exposure based on the estimated exposure ratio map, which could reduce the color brightness distortion.

Although the above methods have achieved decent results in some image enhancement tasks, the performance is heavily dependent on the selection of model parameters, which limits the application on varied scenarios. To improve generalizability, Retinex-based deep learning methods have been increasingly used in low-light image enhancement tasks. In 2017, Shen et al. proposed MSRNet [11] to transform the MSR model into a feedforward convolutional neural network that could directly learn the end-to-end mapping of dark and bright images, but it was weak in noise suppression. RetinexNet [12] proposed by Wei et al. in 2018 firstly decomposes the input image by DecomNet, and then used EnhanceNet to realize the illumination image light adjustment, finally synthesizes the processed images to get the enhancement result. However, these methods tended to use a consistent denoising module to denoise the full image indiscriminately, and they found it difficult to handle the large differences in reflected illumination regions. In 2019, Zhang et al. proposed KinD [19] to eliminate the degradation effect of reflection image by RestorationNet and adjust the light intensity of illumination image by AdjustmentNet. Later, KinD++ [20], released in 2020, presented a novel multi-scale illumination attention module (MSIA), which not only allowed targeted denoising according to the lighting conditions in different regions, but also effectively solved the color distortion problem. In 2021, Chen et al. proposed an up-sampling algorithm [21] for single low-light images. The algorithm enhanced the illumination component and up-sampled the reflectance component by two sub-networks, and then fused the illumination and reflectance components based on the image gradient map. The algorithm achieved better results in color reconstruction and texture feature preservation. In 2021, Wang et al. proposed a reversible normalizing flow model LLFlow [24], which mapped the illumination-invariant color distribution of the normal exposure image to a Gaussian distribution, aiming to extract local pixel correlation and global image features. LLFlow had a complex structure, and it could adaptively recover image illumination while suppressing noise and artifacts. In 2022, Ma et al. proposed SCI [18], composed with share-weighted cascaded enhancement modules. SCI could greatly reduce the inference time while ensuring the vivid color of the enhanced output, but it was not capable of strong denoising. In 2023, Fu et al. concluded that the point-wise multiplication operation of the reflection and illumination components amplifies noise in low-light images, so a synthetic neural network module was used instead of the point-wise multiplication operation to obtain enhanced images [25]. They also used contrastive learning and self-knowledge distillation to constrain the network. Cai et al. proposed a transformer-based algorithm Retinexformer [26]. It designed an illumination-guided transformer for the low-light enhancement task, which could direct the modeling of long-range dependencies and interactions of regions with different lighting conditions according to the captured illumination information.

### Direct low-light images enhancement methods

In addition to these Retinex-based methods, some methods do not need to decompose the input image, but rather enhance the original image directly.

In 2011 Dong et al. applied the image defogging algorithm to reversal low-light image to achieve the image enhancement [10]. The distribution of foggy image and reversed low-light image were not exactly the same, which limited the enhancement effect. In 2016, Lore et al. proposed LLNet [13] to attain adaptive low-light enhancement by stacked sparse denoising autoencoder (SSDA). Due to the simple structure, it tended to blur image details. Chen et al. applied a data-driven approach [22] in 2018 to directly train a fully convolutional network with the SID dataset containing low-exposure images and corresponding high-exposure images. In the same year, Wang et al. proposed GLAD [14] that scales the original image and inputs it into a codec network to estimate the global illumination, then fills in the detailed information that was lost during image scaling. GLAD is more effective in recovering overall brightness, but it is easy to cause color distortion.

In 2019, Jiang et al. proposed an unsupervised low-light image enhancement network EnlightenGAN [17], which created unpaired mappings between low-light and normal images, greatly simplifying the reliance on paired datasets. However, EnlightenGAN was hard to accurately recover the backlit regions, which could easily lead to color bias and artifacts. Based on this, our previous work SRANet [27] further improve the supervised training and adversarial training methods so that the algorithm could be trained using both paired and unpaired datasets, and we presented a noise reduction module based on Patch-GAN, which greatly suppressed the noise of unpaired images during the enhancement process. In 2020, MIRNet [23] proposed by Zamir et al. extracts a complementary set of features across multiple spatial scales, which could not only ensure accurate spatial details but also provide strong contextualized representations. Zeng et al. proposed the Image-Adaptive-3DLUT algorithm [15] model by combining 3DLUT with CNN. It was lightweight and realized the enhancement of high-resolution images in real time. Still, this method was less effective in processing high noisy images due to the lack of a denoising module. Guo et al. proposed a Zero-Reference Deep Curve Estimation (Zero-DCE) [16] model. To obtain the best-fit light enhancement curve, they designed an exquisite loss function to iterative curve parameter learning by implicate evaluating each output image quality. Although Zero-DCE has high inference efficiency, it tends to cause edge flares and color distortion. In 2023, Yin et al. proposed a controllable light enhancement diffusion model CLE Diffusion [29], which encoded the illumination information and utilizes the conditional diffusion model to achieve controlled light enhancement of the image. It also introduces the segment-anything model to allow the user to select regions of interest for enhancement.

In conclusion, the excellent performance of neural network has made it the main way to solve the low-light enhancement task, so our proposed DFEN also uses a structure based on convolutional neural networks to realize the enhancement of low-light images.

## Proposed method

**Fig. 1:**
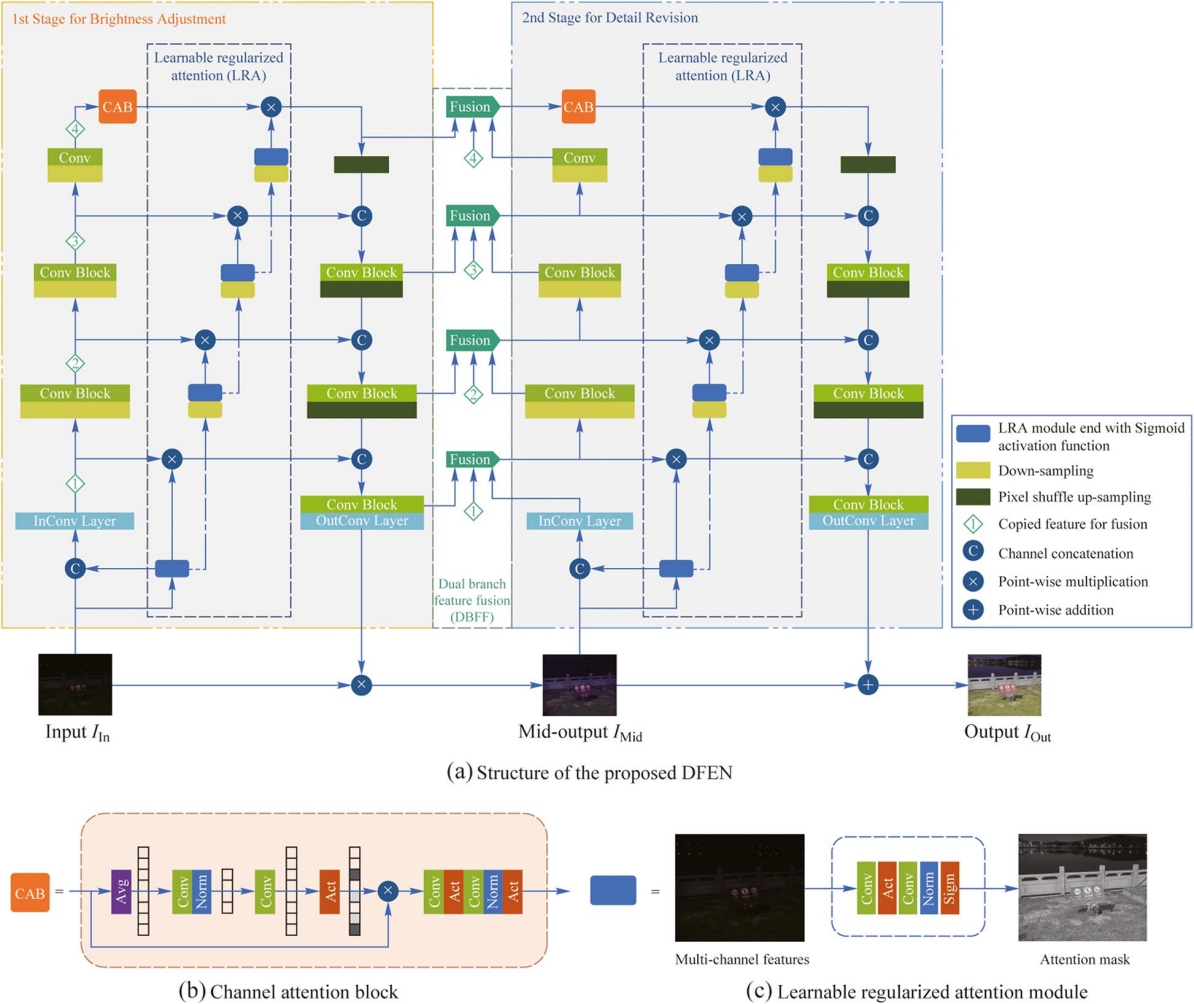
Overall architecture of DFEN. **a** shows the structure of the whole framework, where InConv layer and OutConv layer are 1×1 convolutional layer for dimension transforming. The outcome channels number of the InConv block is noted as the size-controlled hyperparameter, and we obtain the number of channels in each feature layer by multiplying different coefficients on the basic channels. **b** gives the detail of the channel attention block (CAB). **c** displays the structure and samples of the learnable regularized attention (LRA) module

It is challenging to obtain satisfactory results when enhancing brightness and suppressing noise simultaneously. Therefore, we decompose the full enhancement task into two stages to accomplish brightness adjustment and detail revision in sequence. Figure 1 shows the architecture of our proposed end-to-end DFEN and the main notations of the work are formally summarized in Table 1. The low-light input image ${I}_{\text{In}}$ conducts point-wise multiplication with the output of the first stage to obtain the brighten image ${I}_{\text{Mid}}$. This process is equivalent to multiplying each pixel by a luminance enhancement factor to achieve the brightness adjustment of $I_{\mathrm{In}}$. Subsequently, ${I}_{\text{Mid}}$ is used as the input to the second stage and we produce the enhancement result ${I}_{\text{Out}}$ by point-wise addition ${I}_{\text{Mid}}$ with the output of the second stage. This stage adds a detail revision bias to each pixel, aimed for the color correction and denoising of ${I}_{\text{Mid}}$.

**Table 1:** Main notations and descriptions

Notation	Description	Notation	Description
${I}_{\text{In}}$	The input low-light image	${I}_{\text{Ref}}$	The reference image
$I_{{{\text{Mid}}}}$	The middle brighten image	$I_{{{\text{Out}}}}$	The output enhance result
${{Ci}}_{{{i}}} \,$	The input feature streams of channel fusion branch	${C}_{\text{Mid}}$	The spatial global information embedding
${{Cw}}_{{{i}}} \,$	The channel feature fusion weights	$Co$	The channel feature fusion result
${F}_{\text{ex}}$	The channel-upscaling convolution	${F}_{sq}$	The channel-downscaling convolution
${{Si}}_{{{i}}} \,$	The input feature streams of spatial fusion branch	${S}_{\text{Mid}}$	The channel global information embedding
${{Sw}}_{{{i}}} \,$	The spatial feature fusion weights	${So}$	The spatial feature fusion result
${F}_{\text{Adj}}$	The brightness adjustment net	${F}_{\text{Riv}}$	The detail revision net

Our proposed DFEN applies a dual U-Net structure with a channel attention block (i.e., SE block [30]) to extract multi-scale features, which enables the model to learn richer contextual information from the input images [31, 32]. Subsequently, we adopt a dual branch feature fusion (DBFF) module that highlights the key feature information in the channel and spatial dimensions by weighted fusion, thus enhancing the color restoration and detail preservation ability of the model. In addition, we design a learnable regularized attention (LRA) module to fit the lighting condition of the low-light images and guides the model to balance the enhancement effects for different regions. Finally, the cosine training strategy is introduced to gradually adjust the loss weights of the two-stage network, which leads to smoother transition between the two-stage tasks and achieves better integrated enhancement results.

### Dual branch feature fusion module

**Fig. 2:**
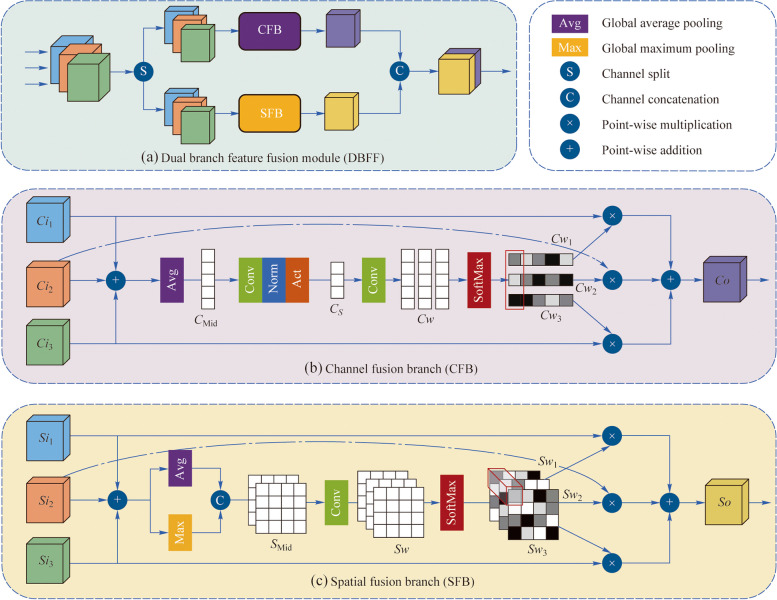
Structure of the dual branch feature fusion module

In order to compensate for the high-resolution information lost during the scale transformation of the U-Net and shorten the feature path of the network, we adopt the dual branch feature fusion (DBFF) module shown in Fig. 2 in the encoder part of the second-stage to perform weighted fusion of the same scale features in the encoder and decoder parts of the two-stage network.

Inspired by the dual attention branch designed in SANet [33], DBFF splits the multiple input feature streams in the channel dimension, and parallel performs feature adaptive selection and aggregation in channel fusion branch (CFB) and spatial fusion branch (SFB). Finally, we concatenate the output of the two branches to obtain better fusion feature representations.

In the channel fusion branch, we first perform point-wise addition on the multiple input feature streams $\left\{{Ci}_{1}, {Ci}_{2}, {Ci}_{3}\right\}\;\in\;{R}^{H\times W\times C}$, and then obtain ${C}_\text{Mid}\;\in\;{R}^{1\times 1\times C}$ by applying global average pooling, which embeds the spatial global information of input features. Subsequently, we obtain the inter-channel relationships through squeeze and excitation operations and generate the channel fusion weights *Cw*_1_, *Cw*_2_ and *Cw*_3_.

1 $$C_{\mathrm{Mid}}=\mathrm{GAP}(C_{\mathrm{add}})=\mathrm{GAP}(Ci_1+Ci_2+Ci_3),$$

2 $$Cw = F_{{{\text{ex}}}} (Cs) = F_{{{\text{ex}}}} (F_{{{\text{sq}}}} (C_{{{\text{Mid}}}} )),$$

where *F*_sq_ is a channel-downscaling convolution layer and *F*_ex_ is a channel-upscaling convolution layer. $\left\{{Cw}_{1}, {Cw}_{2}, {Cw}_{3}\right\}\;\in\;{R}^{1\times 1\times C}$ is split from *Cw*. $Cw\;\in\;{R}^{1\times 1\times 3C}$, $Cs\;\in\;{R}^{1\times 1\times \frac{C}{4}}$ is the typical setting in our method.

Finally, we compute the fusion result of the channel dimension according to Eq. (3). Note that the SoftMax function is used to normalize the weights ${\alpha }_{c}$, ${\beta }_{c}$, ${\gamma }_{c}$ of the same channel in *Cw*_1_, *Cw*_2_, *Cw*_3_, which makes ${\alpha }_{c}+{\beta }_{c}+{\gamma }_{c}=1$.

3 $$Co=Cw_1\cdot Ci_1+Cw_2\cdot Ci_2+Cw_3\cdot Ci_3.$$

Spatial fusion branch is similar to the channel fusion branch. After point-wise add $\left\{{Si}_{1}, {Si}_{2}, {Si}_{3}\right\} \in {R}^{H\times W\times C}$, we perform global average pooling and maximum pooling along the channel dimensions and concatenate the results to obtain ${S}_{\text{Mid}}\in {R}^{H\times W\times 2}$. Then, we generate the spatial fusion weights $\left\{{Sw}_{1}, {Sw}_{2}, {Sw}_{3}\right\} \in {R}^{H\times W\times 1}$ via a convolution layer with a kernel size of 3. Finally, we implement the spatial feature fusion based on the normalized *Sw*_1_, *Sw*_2_ and *Sw*_3_.

4 $$S_{\mathrm{add}}=Si_1+Si_2+Si_3,$$

5 $$S_{\mathrm{Mid}}=\mathrm{Concat}\left(\mathrm{GAP}\left(S_{\mathrm{add}}\right),\mathrm{GMP}\left(S_{\mathrm{add}}\right)\right),$$

6 $$So=Sw_1\cdot Si_1+Sw_2\cdot Si_2+Sw_3\cdot Si_3.$$

DBFF reconstructs the input feature streams in the channel and spatial dimensions, which effectively compensates for the high-resolution detail information lost due to the network layer deepening, thus enhancing the color recovery and detail preservation capabilities of the DFEN. Moreover, the adaptive generation of fusion weights through global pooling and convolution operations can more accurately guide the model to emphasize the significant features of inputs streams.

### Learnable regularized attention module

In our previous study, the quality of enhancement can be significantly improved by adding a simple self-regularized attention map, which allows the model to enhance both light and dark areas of the image appropriately [27]. However, we find that when using fixed coefficients to obtain an attention map, the contrast of the image may be lost, making it difficult to accurately distinguish regions of similar brightness but different colors, which results in color distortion in the enhanced images.

To solve this problem, we replace the fixed coefficients with an LRA module to acquire the single-channel attention map. We then concatenate it with the input image and send them to the InConv layer together. At the same time, the attention maps are downsampled progressively using the same downsampling method as in the U-Net structure in order to adapt to different feature scales. In addition, the attention maps will also be multiplied with the features in the skip connections of the U-Net for better guidance. It is worth noting that the last activation of each attention block is sigmoid to limit the value range from 0 to 1. Through the addition of the LRA module, DFEN can effectively suppress the overexposure in bright regions and adaptively balance the enhancement performance of different regions, which will be verified in the ablation experiments.

### Cosine training strategy

**Fig. 3:**
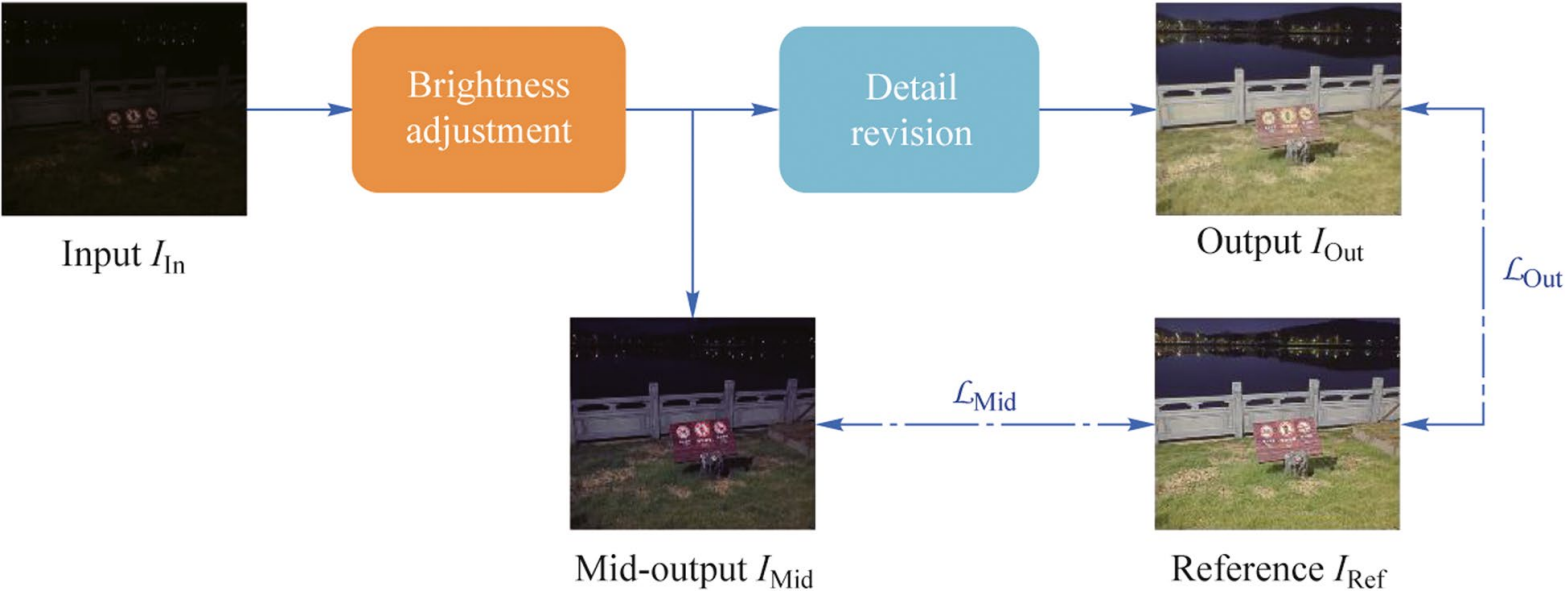
Loss function configuration of DFEN

As illustrated in Fig. 3, we establish distinct loss functions for the mid-output and output images to the reference image. This makes DFEN focus on different tasks during the different epochs of training. Besides, we propose a cosine training strategy that dynamically adjusts the weights of the loss function during training to make the transition between the two task stages smoother.

When training DFEN, we adopt L1 loss and SSIM loss [34] between the output image $I_{{{\text{Out}}}}$ and the reference image $I_{{{\text{Ref}}}}$ to accurately restore the details. Besides, the content loss is computed as an addition to constrain the perceptually similar [35]. The loss of the output image is as follows:

7 $${\mathcal{L}}_{{{\text{Out}}}} \left( {I_{{{\text{Out}}}} ,I_{{{\text{Ref}}}} } \right) = w_{1} {\mathcal{L}}_{1} + w_{{{\text{SSIM}}}} {\mathcal{L}}_{{{\text{SSIM}}}} + w_{{{\text{Cont}}}} {\mathcal{L}}_{{{\text{Cont}}}},$$

where $w_{1}$, $w_{{{\text{SSIM}}}}$ and $w_{{{\text{Cont}}}}$ are the weight of ${\mathcal{L}}_{1}$, ${\mathcal{L}}_{{{\text{SSIM}}}}$ and ${\mathcal{L}}_{{{\text{Cont}}}}$, respectively.

We also apply the loss function directly between the mid-output $I_{{{\text{Mid}}}}$ and the reference image $I_{{{\text{Ref}}}}$ as follows:

8 $$\mathcal{L}_{\mathrm{Mid}}\;\left(I_{\mathrm{Mid}},\;I_{\mathrm{Ref}}\right)\;=\;w_1^{\prime}\mathcal{L}_1^{\prime}\;+\;w_{\mathrm{SSIM}}^{\prime}\mathcal{L}_{\mathrm{SSIM}}^{\prime}.\\$$

As the pseudo-code shown in Algorithm 1, the training of DFEN is divided into two stages. During the first stage, we compute all the losses after one forward propagation to make each parameter get enough gradient. In the second stage, we only calculate ${\mathcal{L}}_{{{\text{Out}}}}$. We add a cosine conversion factor to control the transition between the two stages, and the total loss function of the network is shown as follows:

9 $$\left\{ \begin{gathered} {\mathcal{L}}_{{{\text{Total}}}} = c \times {\mathcal{L}}_{{{\text{Mid}}}} + \left( {1 - c} \right) \times {\mathcal{L}}_{{{\text{Out}}}} , \hfill \\ c = \max \left( {\cos \left( {\uppi \times \frac{{{\text{Epoch}}}}{N}} \right),0} \right), \hfill \\ \end{gathered} \right.$$

where *c* is a coefficient that satisfies cosine descent until 0 throughout the training process. Epoch represents the current number of training epochs $N$ is the total training epochs.

**Figure:**
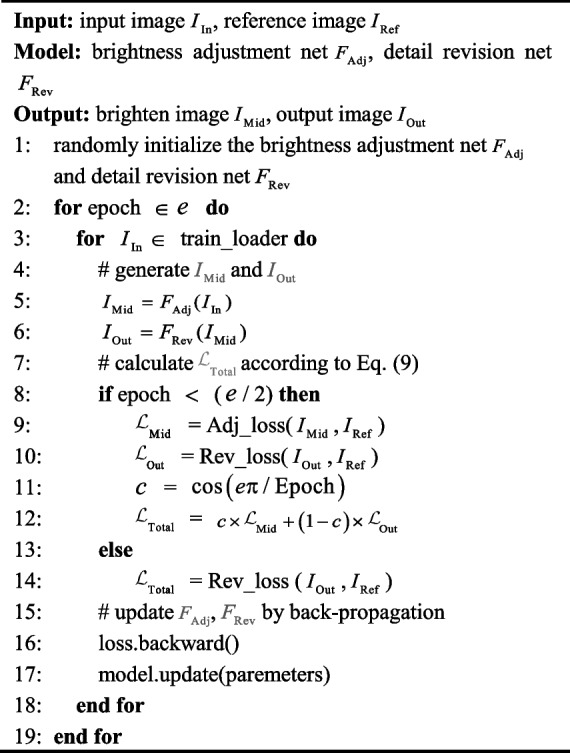
**Algorithm 1** Training of DFEN

## Experimental results and discussion

In this section, we conduct comparison and ablation experiments to reveal the advance of the proposed DFEN.

### Datasets description

**Fig. 4:**
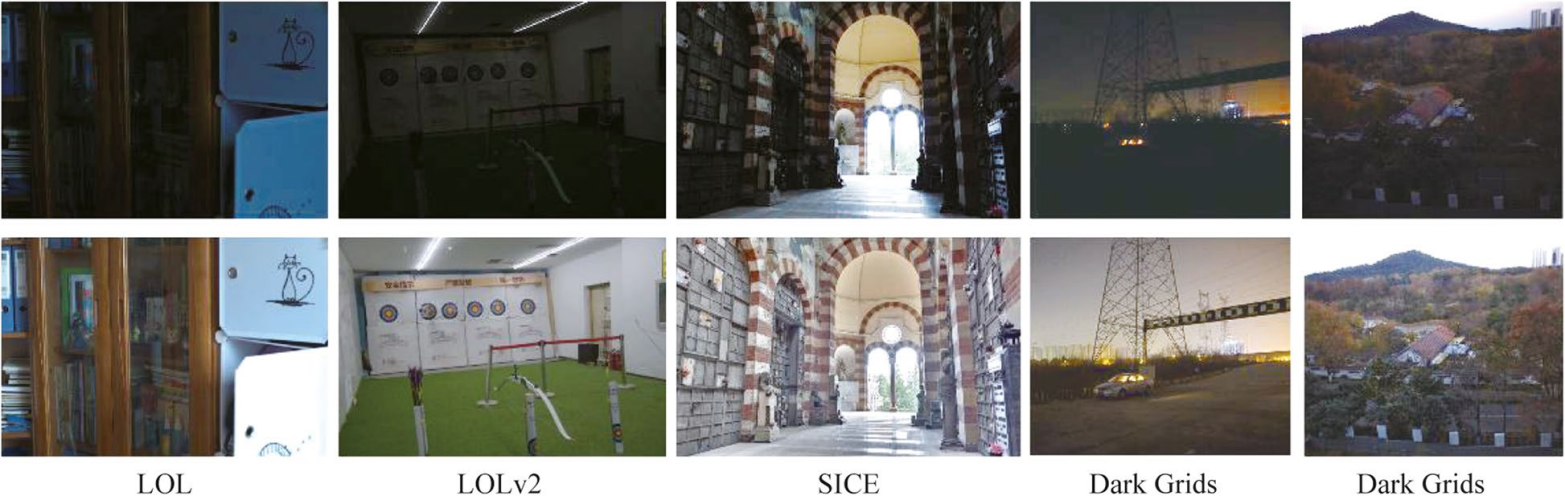
Samples of each dataset. The images in the first row are low-light inputs, while the images in the second row are the references

Public datasets LOL [12], LOLv2 [36] and SICE [37] are selected for comparison experiments. The details of each dataset are shown in Table 2, and some reference samples are shown in Fig. 4.

**Table 2:** Details of each datasets

Datasets name	Training	Testing	Size	Total megapixels
LOL	485	15	600×400	0.24
LOLv2	689	100	600×400	0.24
SICE	531	58	Various^a^	0.36
Dark Grids	530	103	1224×1024	1.25

^a^Width is from 692 to 843, while height is from 426 to 519, in total 0.36 megapixels per image

For the low-light power inspection scenes of the project, there is currently no public dataset of that can be used for enhancement model training and evaluation. So, we constructed the Dark Grids dataset using inspection imaging equipment, which mainly consists of nighttime transmission tower scenes, high-dynamic-range scenes and daytime normal exposure scenes.

We use a 12-bit industrial camera to capture images from different times and locations. For each scene, we first capture a long-exposure (512 ms) image *N*_1_ and then take the image sequence with different exposures from 1 to 512 ms step by step. Another long-exposure (512 ms) image *N*_2_ is taken at the end. We calculate the MSE metrics between *N*_1_ and *N*_2_, and filter out the sequences of images with high similarity (>0.98). Next, we applied the multi-frame HDR fusion algorithm in Adobe Photoshop CC to obtain the reference image of each scene. Finally, we collected a total of 530 pairs of training samples and 103 pairs of test samples with 1224 × 1024 pixels.

### Implementation details

The entire algorithm is built on the PyTorch framework. As mentioned in Fig. 1, the proposed DFEN can quickly change its size by setting different channel numbers of InConv layer. To evaluate the enhancement effect of different volume models, we set different sizes of DFEN as 8, 16, 24, corresponding to DFEN-s, DFEN-m and DFEN-l, respectively.

When training DFEN, random flip, affine transform and random crop are used to enhance both low-light and reference images to obtain 512 × 512 image pairs. We tuned the hyperparameters by manual tuning and grid search. The multiplication coefficients of each feature layer of U-Net are set to 1, 4, 16 and 32 and different loss weights are set according to $2w^{\prime}_{1} = 2w^{\prime}_{{{\text{SSIM}}}} = w_{1} = w_{{{\text{SSIM}}}} = 10w_{{{\text{Cont}}}} = 1$. Based on experience, AdamW [38] is used as the optimizer and the batch size is set to 12. A total of 600 epochs are trained to avoid overfitting.

### Quantitative comparison experiments

We focus the comparison experiment on the proposed DFEN with thirteen mainstream low-light image enhancement methods, including three traditional methods LIME [7], BIMEF [8], CRM [9], and ten deep learning methods RetinexNet [12], GLAD [14], MIRNet [23], EnlightenGAN [17], KinD++ [20], Zero-DCE++ [39], Adapt-3DLUT [15], SCI [18], LLFlow [24], SRANet [27]. It is worth mentioning that for the unpaired training part involved in SRANet and EnlightenGAN, the low-light and the corresponding reference images are shuffled separately and randomly collected to form the unpaired training batch.

**Fig. 5:**
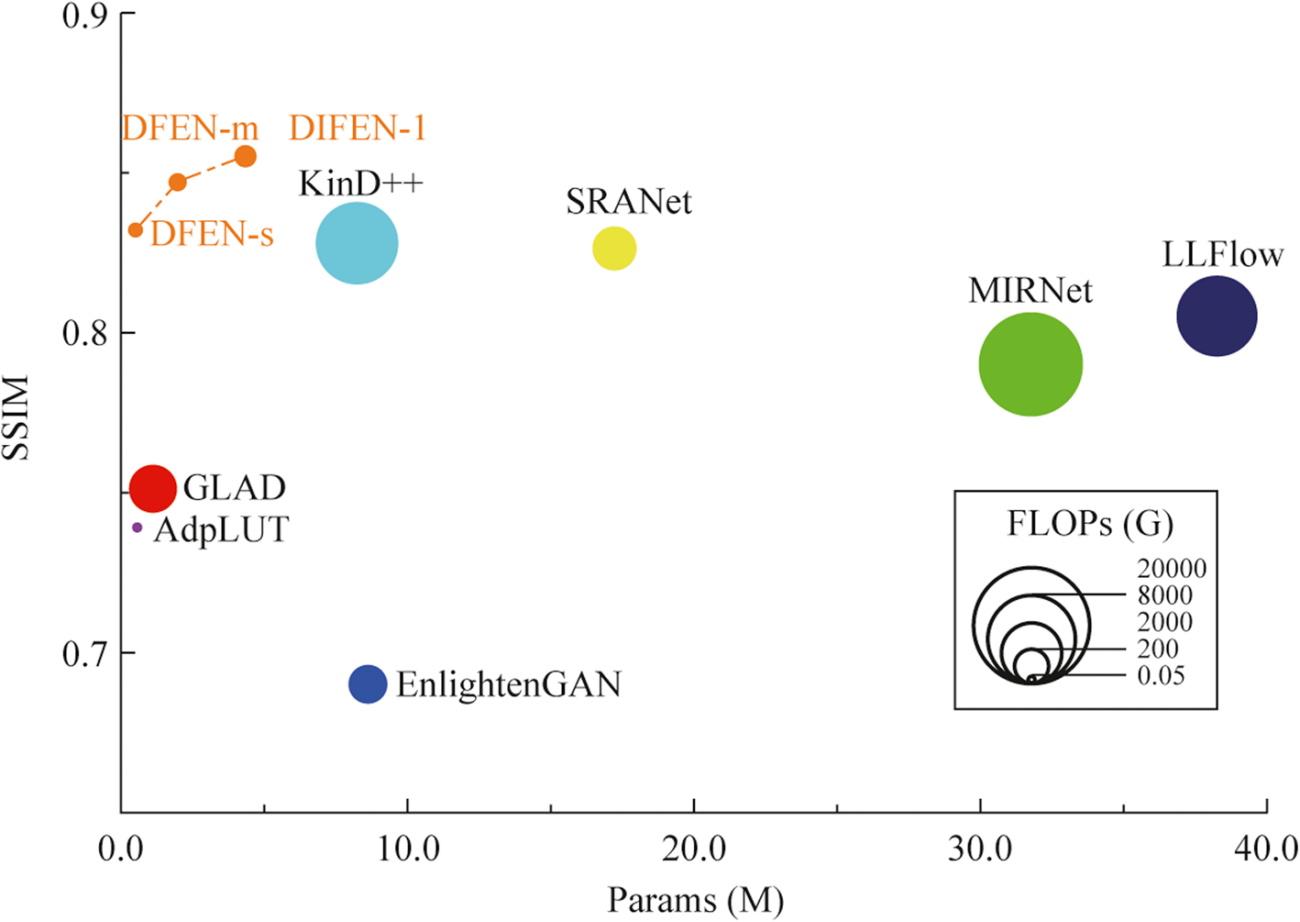
SSIM metric in the LOLv2 dataset and computational efficiency (Params and FLOPs) of each comparison algorithm

**Fig. 6:**
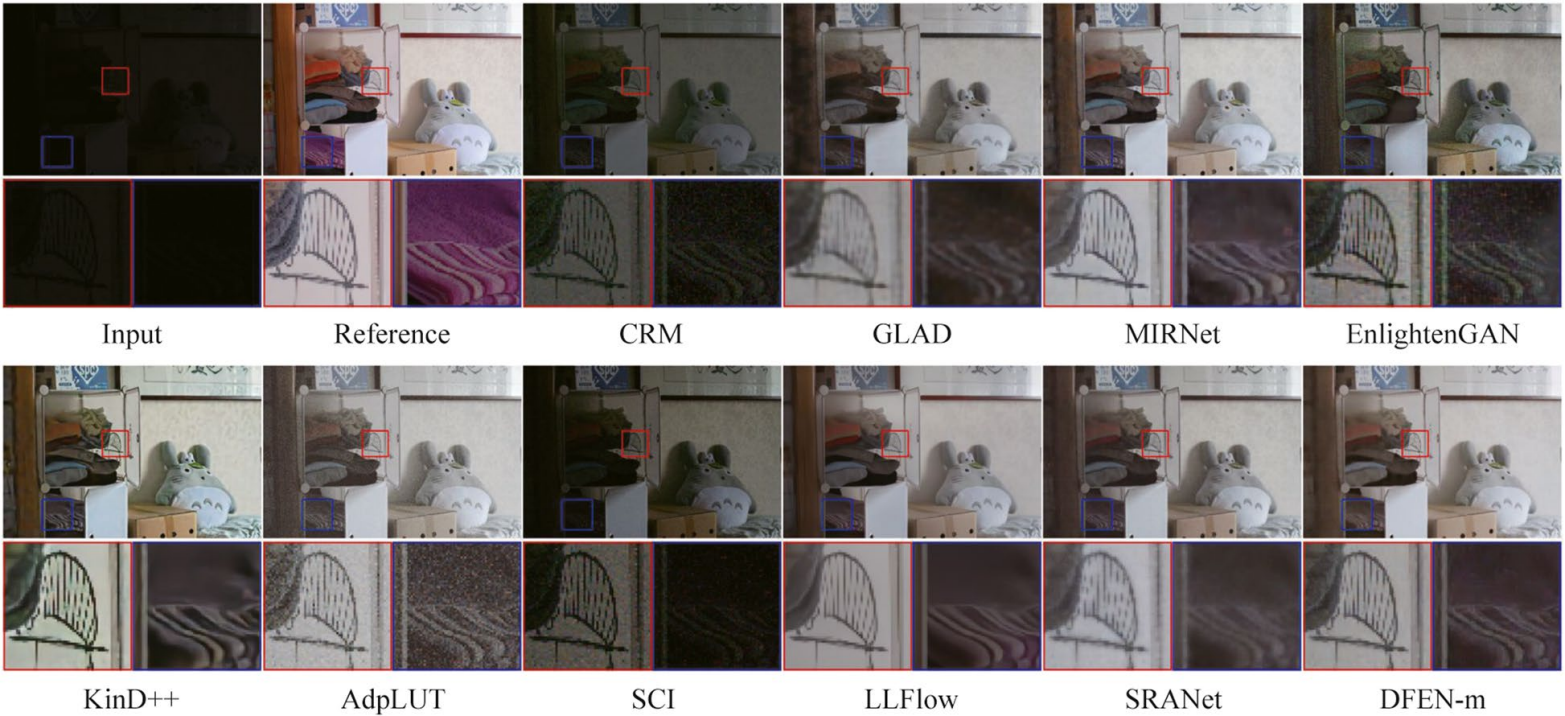
Visual comparisons of DFEN and other methods on LOL dataset

**Fig. 7:**
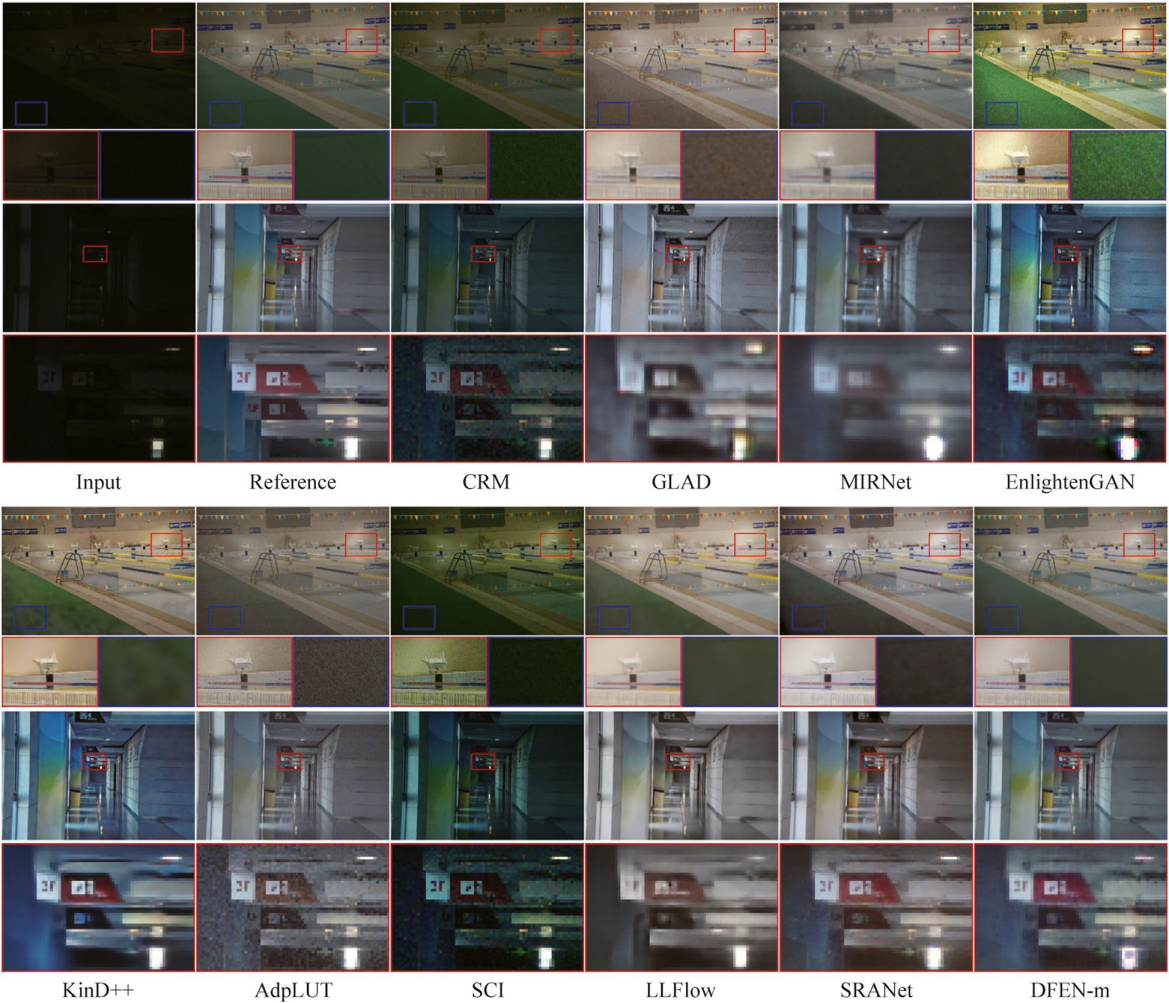
Visual comparisons of DFEN and other methods on LOLv2 dataset

**Fig. 8:**
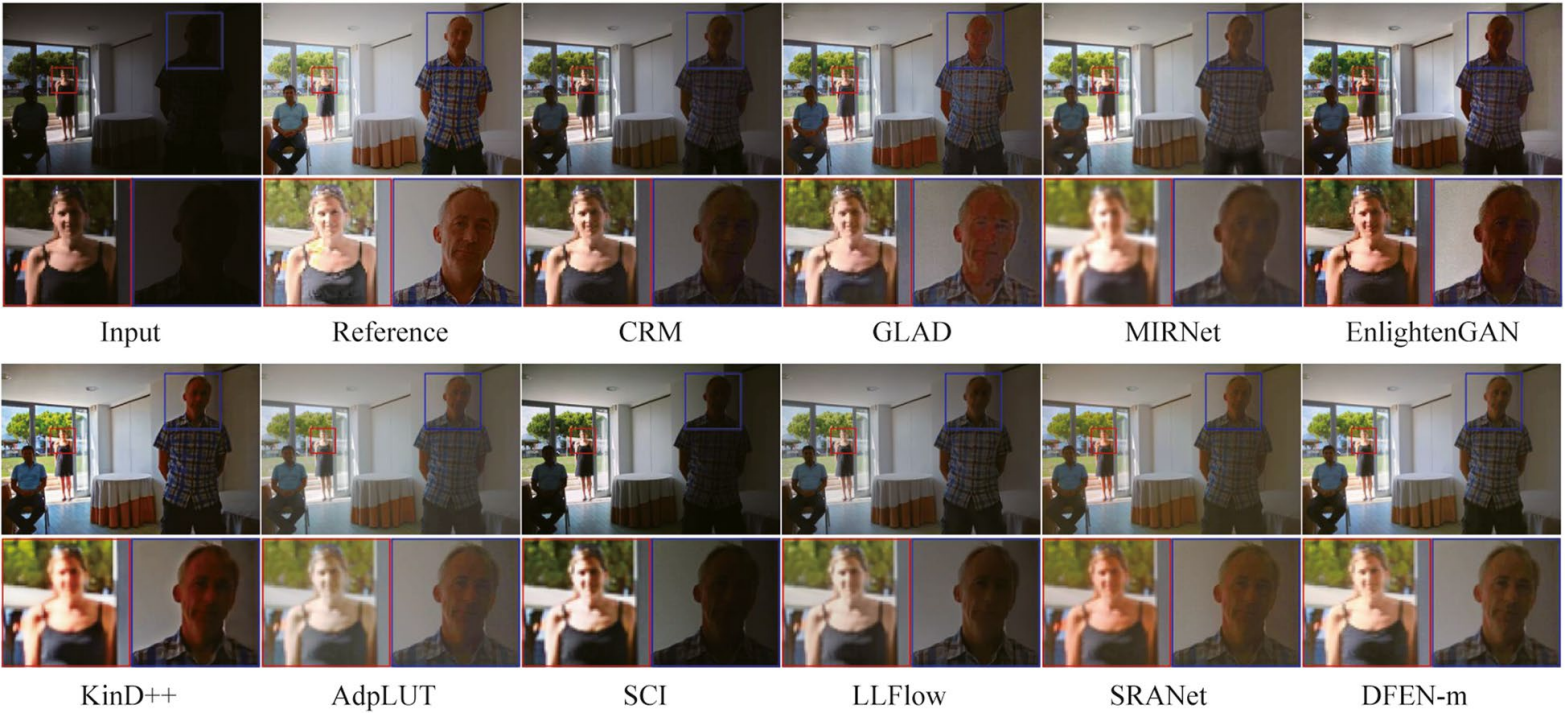
Visual comparisons of DFEN and other methods on SICE dataset

**Fig. 9:**
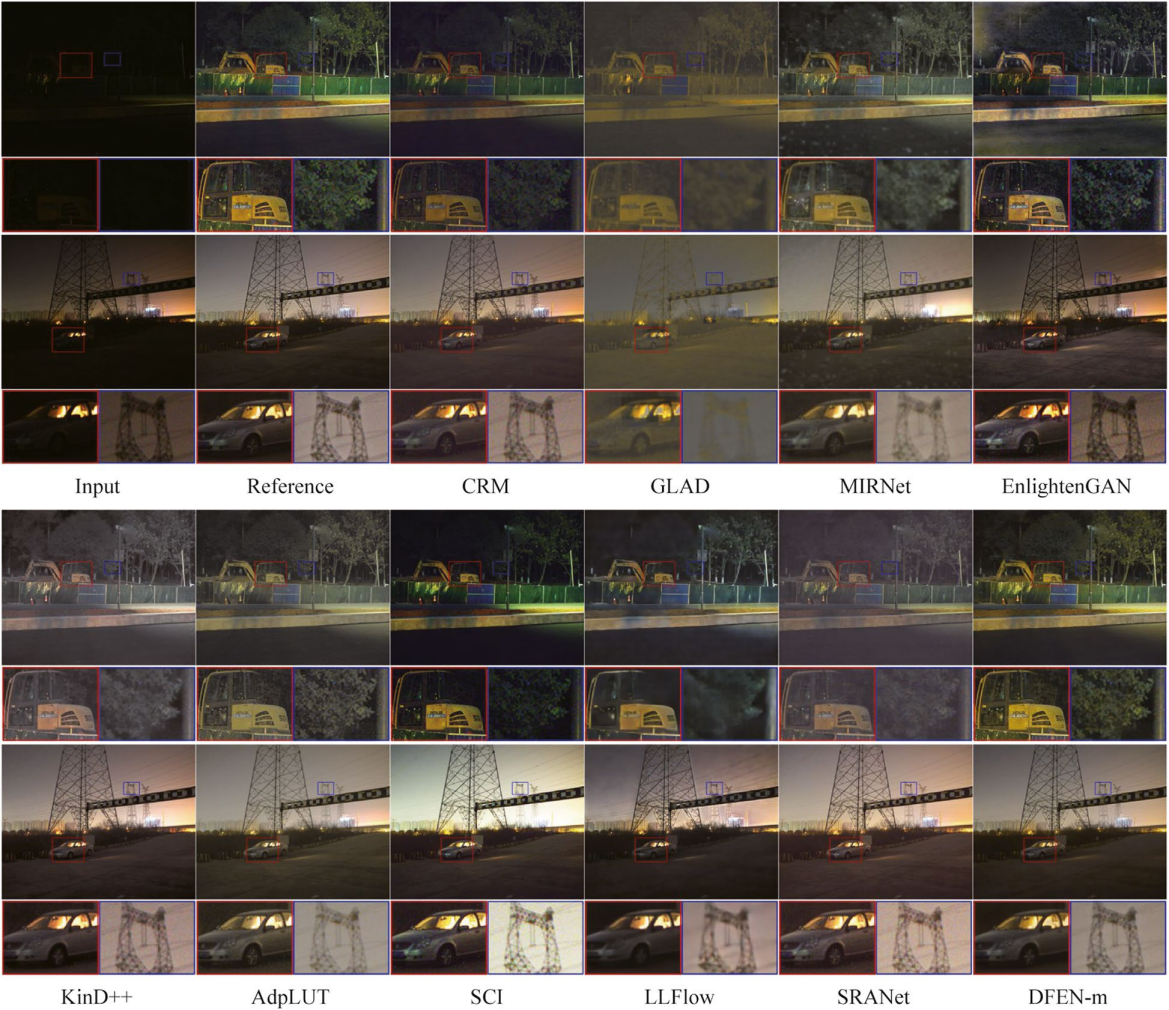
Visual comparisons of DFEN and other methods on our Dark Grids dataset

We compute SSIM [34] and PSNR between the enhanced image and the reference image as quantitative metrics of enhancement performance. Moreover, we use LOEref [19, 40] to evaluate the ability of the algorithm in preserving the naturalness of lightness. Table 3 reports the results, and we also plot the scatter diagram of the SSIM metric and the computational efficiency (Params and FLOPs) for part of the CNN-based algorithms in the LOLv2 dataset, as shown in Fig. 5. Some algorithms with the number of parameters larger than 40 M are not indicated in the figure. Moreover, some enhanced samples of the top 10 comparison algorithms in terms of average SSIM metric are shown in Figs. 6, 7, 8 and 9.

**Table 3:** Results of comparison experiment

Datasets	Metric	Input	LIME	BIMEF	CRM	Retinex Net	GLAD	MIRNet	Enlighten GAN	KinD++	Zero-DCE++	AdpLUT	SCI	LLFlow	SRANet	DFEN-s	DFEN-m	DFEN-l
	Params					0.445M	0.932M	31.787M	8.640M	8.270M	10.564K	0.594M	258	38.860M	17.260M	0.561M	2.130M	4.710M
	FLOPs (G)					67.25	1084.45	15011.9	316.8	6158.23	13.48	0.07	0.33	5377.44	667.84	3.19	9.6	19.38
	Time (ms)		475.30	505.40	521.40	187.43	156.95	7854.28	155.33	1759.32	3.23	2.07	5.75	955.24	133.24	89.50	128.78	171.60
LOL	SSIM↑	0.191	0.445	0.595	0.623	0.425	0.768	*0.842*	0.714	0.828	0.477	0.675	0.511	**0.852**	0.807	0.805	0.823	0.826
	PSNR↑	7.773	16.759	13.875	17.203	16.774	20.108	**24.138**	18.372	21.804	16.704	21.158	16.225	21.133	20.565	21.774	22.060	*23.074*
	LOE_ref_↓	305.716	457.597	323.969	303.424	722.815	337.311	242.569	512.956	324.841	608.809	294.687	332.927	331.863	272.535	271.258	*242.285*	**233.320**
LOLv2	SSIM↑	0.196	0.419	0.639	0.639	0.407	0.751	0.790	0.690	0.828	0.386	0.739	0.532	0.805	0.826	0.832	*0.847*	**0.855**
	PSNR↑	9.718	15.242	17.855	19.655	16.097	18.201	21.695	16.645	20.767	15.592	21.821	17.409	18.337	20.671	21.300	*22.284*	**22.786**
	LOE_ref_↓	262.803	453.532	286.328	268.084	740.086	331.495	309.807	600.801	315.151	647.983	274.525	269.038	313.826	294.184	227.887	*221.515*	**216.516**
SICE	SSIM↑	0.436	0.715	0.753	0.792	0.686	0.793	0.813	0.748	0.800	0.451	0.780	0.709	0.821	0.790	0.818	*0.823*	**0.824**
	PSNR↑	10.060	16.802	16.883	19.873	16.709	20.842	21.606	18.486	20.123	16.287	20.204	17.198	21.349	19.994	22.102	*22.107*	**22.321**
	LOE_ref_↓	456.818	548.104	437.769	495.407	973.143	455.399	417.062	621.292	435.515	744.044	464.118	442.494	440.246	443.667	398.093	**381.098**	*395.394*
Dark Grids	SSIM↑	0.133	0.197	0.273	0.288	0.355	0.412	0.528	0.330	0.450	0.208	0.407	0.238	0.566	0.538	0.561	*0.574*	**0.589**
	PSNR↑	11.216	12.695	14.460	14.485	13.634	15.065	18.793	12.700	16.590	13.699	18.543	12.909	20.804	19.983	21.279	*21.830*	**23.101**
	LOE_ref_↓	814.078	1020.914	895.976	826.115	1279.440	734.339	456.533	1308.341	447.02	1021.497	615.019	814.568	453.308	410.919	390.886	*382.737*	**369.957**

The bolded font is the best score and the italicized underlined font is the second best. ↑/↓ denotes larger/smaller values lead to better quality.

As for the LOL dataset, some networks use weights provided by the original authors. There are only 15 images in the LOL testing set. Our DFENs achieve better enhancement performance in models with a similar number of parameters and obtained the lowest LOE metrics. As shown in Fig. 6, CRM, GLAD and SCI fail to reach an acceptable brightness range and the contrast of the enhanced image is low. AdpLUT and EnligtenGAN have poor denoising ability and the enhanced image contains a lot of noise. It is evident that only the proposed DFEN, as well as MIRNet, Kind++, LLFlow and SRANet with a huge number of parameters, are able to accurately restore the color of the clothes in the plastic box. Among them, Kind++’s result are heavily color biased, while the results of MIRNet and SRANet have blurred details.

For the LOLv2 and SICE datasets, all the networks are retrained, and the proposed DFENs achieve superior results on SSIM, PSNR and LOE. Observing the visualization results, CRM has acceptable noise suppression and color restoration capabilities, but the enhancement effect is poor for dark scenes, making it difficult to recognize the facial details of the people in the dark parts of the image in Fig. 8. GLAD struggles to recover color information from low-light images, and the green carpet next to the swimming pool in Fig. 7 degrades to brown. The structure of MIRNet is too deep to accurately restore the high-frequency features in the shallow layers, resulting in blurred detail. Since EnlightenGAN adopts unsupervised training strategy, it poses a challenge to balance the enhancement effect of different regions, which leads to overexposure and high saturation in the output image. As can be seen in the detail zoomed image, the output of AdpLUT is still noisy due to the lack of a denoise module. Although KinD++ can suppress the noise better, the details are partially lost, and artifacts are generated in the extremely dark regions in the bottom left corner of the first image in Fig. 7. The model of SCI is too simple, resulting in poor enhancement effect and serious color deviation in the enhanced image. LLFlow can effectively suppress the generation of noise and artifacts at the same time, but the model is too complex, leading to a longer inference time. Compared with our previously proposed SRANet, the DFEN redesigns the algorithm structure, introducing the LRA module and novel DBFF module, so that it can restore the image more realistically. Compared with our previously proposed SRANet, the DFEN redesigns the algorithm structure, introducing the LRA module and novel DBFF module, so that it can restore the image more realistically. For the low-light images in Fig. 7, DFEN obtains enhancement results with more realistic colors and clearer details. And for the high dynamic range sample in Fig. 8, DFEN can avoid the over-exposure of bright regions while correctly reveal details in dark regions.

In the Dark Grids dataset, it is easy to find that the proposed method has obvious advantages over other methods. From the displayed samples in Fig. 9, GLAD, KinD++, and SRANet have deficiencies in brightness recovery, making the enhancement results of the first image grayish and the details of the construction vehicles blurred. The enhanced images of CRM, EnlightenGAN and AdpLUT are bright enough but the saturation is low. SCI improved color recovery, but the second image has an over-exposure problem in the car interior. The results of MIRNet and LLFlow lose a great deal of high-resolution detail, leading to severe blurring of the distant towers in the second image. We can see that the DFEN model can solve the problems of color deviation and uneven exposure more accurately, performing better in noise suppression and detail retention. It indicates that DFEN is more suitable for the project’s low-light power inspection scenes than existing algorithms.

Overall, in contrast to other comparative algorithms, the proposed DFEN is more effective in recovering the color and texture details of low-light images. At the same time, DFEN can balance the enhancement effect in different lighting conditions to avoid color distortion and over-exposure in the enhanced image. Moreover, DFEN has achieved outstanding enhancement results in several datasets, which also proves its excellent scene adaptation ability. Although LLFlow shows stronger color recovery and denoising ability in some datasets, DFEN has the advantage of computational efficiency, which is more suitable for the scenarios of our project.

### Ablation study

To validate the necessity of each modules in the proposed method, we design several ablation experiments, configuration and results are shown in Tables 4 and 5. Note that the quantitative evaluation of all ablation experiments was performed on the LOLv2 dataset and we still use SSIM, PSNR and LOE_ref_ as quantitative evaluation metrics. We consider DFEN-m as the baseline algorithm. Except for the modules evaluated, all the experiments share the identical experimental setups.

**Table 4:** Results of structure ablation experiment

No.	Ablation description	SSIM↑	PSNR↑	LOE_ref_↓
A1	DFEN w/o DBFF	0.802	19.589	258.381
A2	DFEN with concatenation fusion	0.824	21.373	236.389
A3	DFEN only with spatial fusion	0.815	20.281	227.467
A4	DFEN only with channel fusion	*0.837*	21.560	**212.837**
B1	DFEN w/o LRA module	0.833	*21.861*	226.030
B2	DFEN with fixed SRA module	0.821	21.736	244.342
DFEN-m	Proposed DFEN-m	**0.847**	**22.284**	*221.515*

The bolded font is the best score and the italicized underlined font is the second best. ↑/↓ denotes larger/smaller values lead to better quality

**Table 5:** Results of training strategy experiment

No.	${\mathcal{L}}_{\mathrm{SSIM}}^{'}$	${\mathcal{L}}_{\mathrm{1}}^{'}$	${\mathcal{L}}_{{{\text{SSIM}}}}$	${\mathcal{L}}_{1}$	${\mathcal{L}}_{{{\text{Cont}}}}$	Cos	SSIM↑	PSNR↑	LOE_ref_↓
C1				√		√	0.818	21.912	254.423
C2				√	√	√	0.837	21.875	235.509
C3			√	√		√	0.840	22.173	226.409
C4			√	√	√	√	0.821	22.229	**216.429**
C5		√		√		√	0.828	22.092	238.920
C6		√		√	√	√	0.838	**22.338**	231.859
C7	√	√	√	√		√	0.813	22.110	248.297
C8	√	√	√	√	√		*0.842*	21.434	238.200
DFEN-m	√	√	√	√	√	√	**0.847**	*22.284*	*221.515*

The bolded font is the best score and the italicized underlined font is the second best. ↑/↓ denotes larger/smaller values lead to better quality.

#### Ablation experiments of feature fusion strategy

**Fig. 10:**
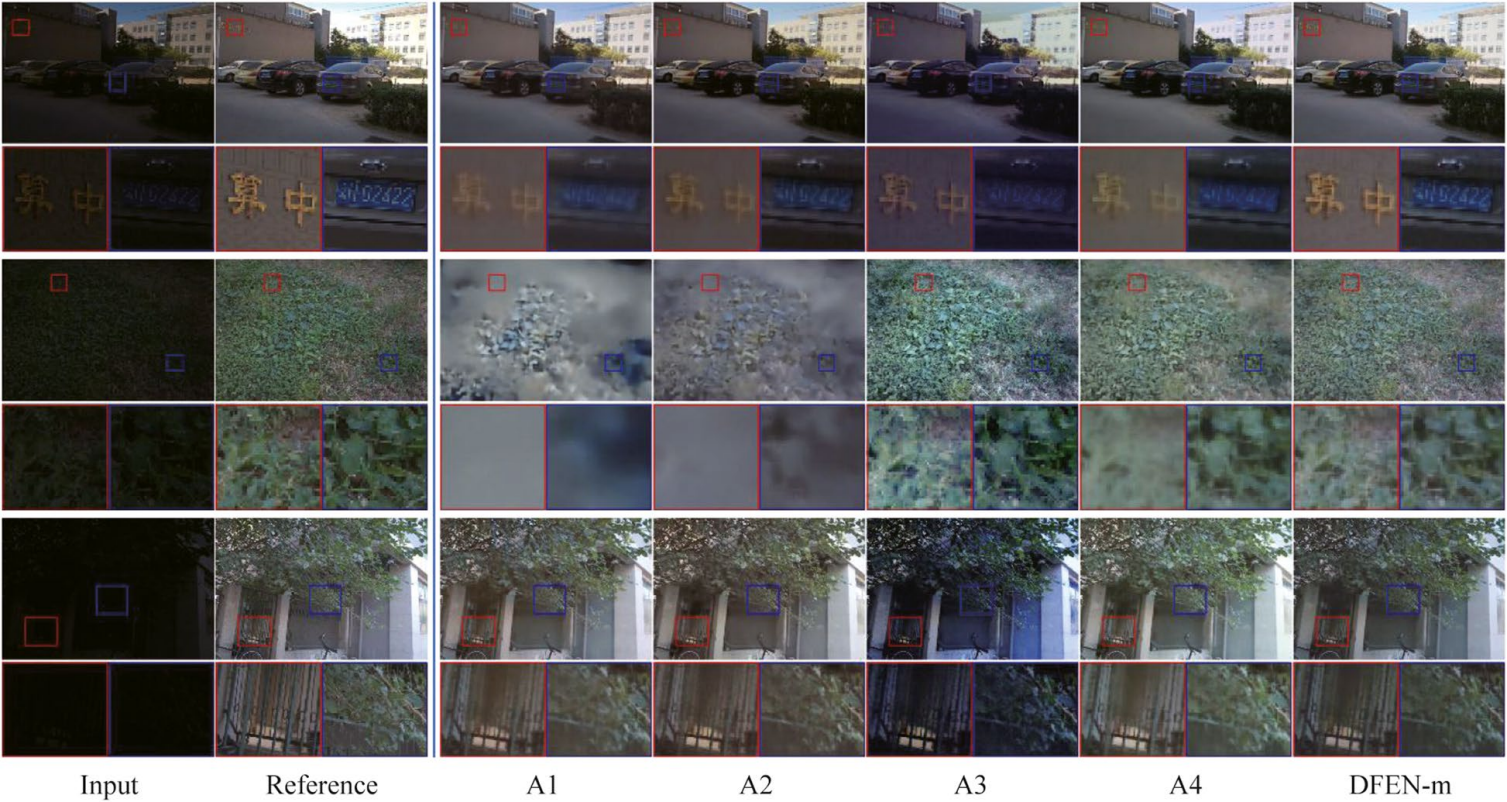
Visual comparisons of different feature fusion strategies

We first conducted ablation experiments for different feature fusion methods, and several enhanced samples are displayed in Fig. 10. A1 removes the feature fusion module between the two stages, the high-resolution features are partly lost after the scale transformation, resulting in serious blurred details in the outputs, as well as the worst evaluation metrics. Compared with the common concatenation fusion in A2, A3 and A4 can highlight the key features of the same channel or spatial position in the feature group by using the spatial or channel dimension weighted fusion method, thus obtaining better texture detail retention and color recovery capabilities, respectively. Observing the enlarged image, it can be seen that the characters, lawns and railings in the enhanced images are clearer when using the spatial fusion module, but there are some color deviations in the whole image. Although the color of the enhanced image obtained by A4 is more realistic, it suffers from a degree of blurring in the details. The DBFF module adopted by DFEN makes the fusion weights of the spatial and channel dimensions to be independent of each other by grouping the feature maps to jointly improve the fusion effect of the model, leading to enhancement results with clear details and realistic colors.

The introduction of the feature fusion module improves the performance of the algorithm, but also increases the computational complexity. The proposed DBFF uses channel splitting to divide the features into two groups to balance the computation and enhancement effect of the algorithm. In practical applications, we can adjust the preferences for either enhancement effect or computation by duplicating the features or grouping them by channel dimensions

#### Ablation experiments of illumination attention module

**Fig. 11:**
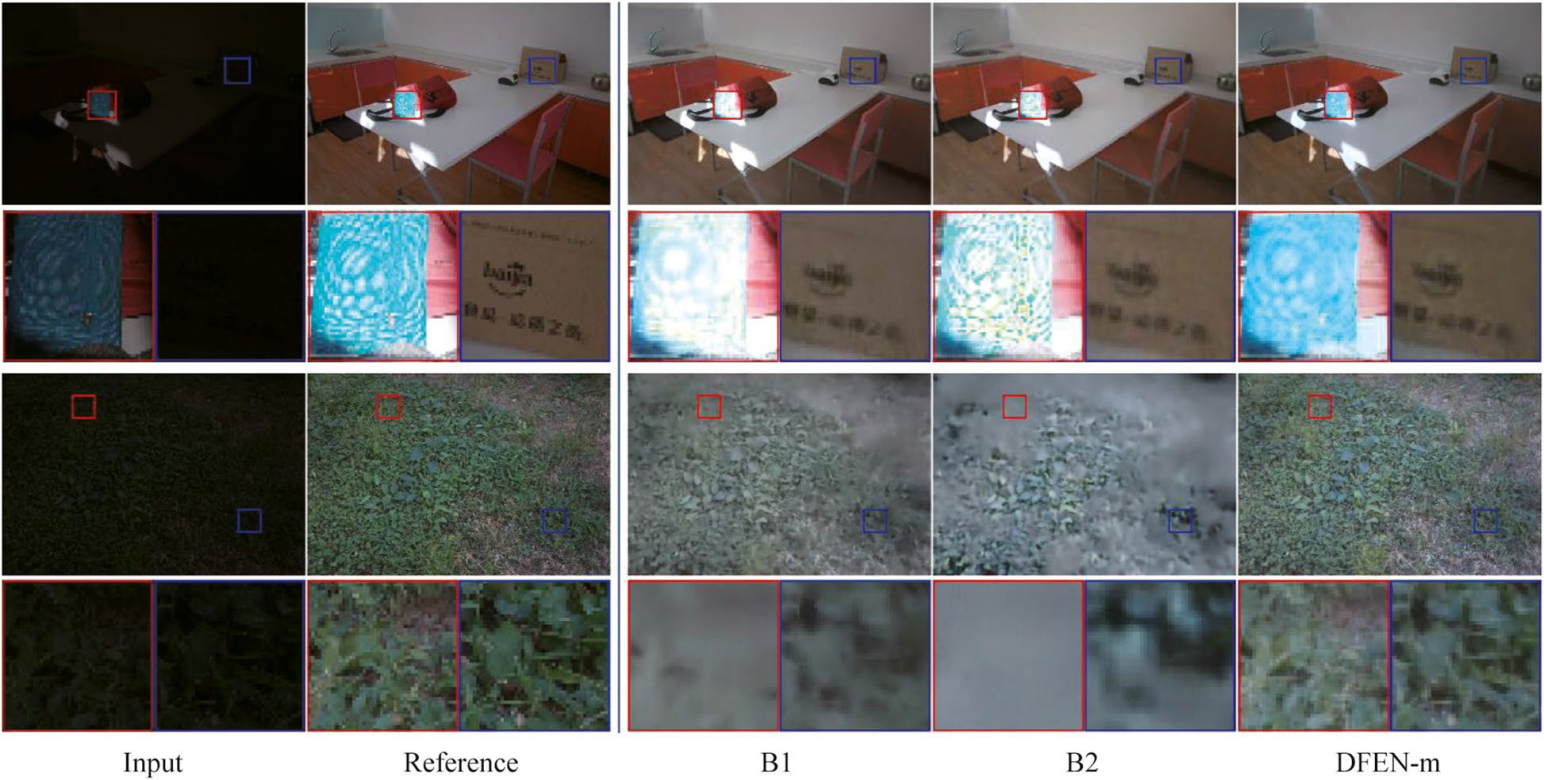
Visual comparisons of different illumination attention modules

**Fig. 12:**
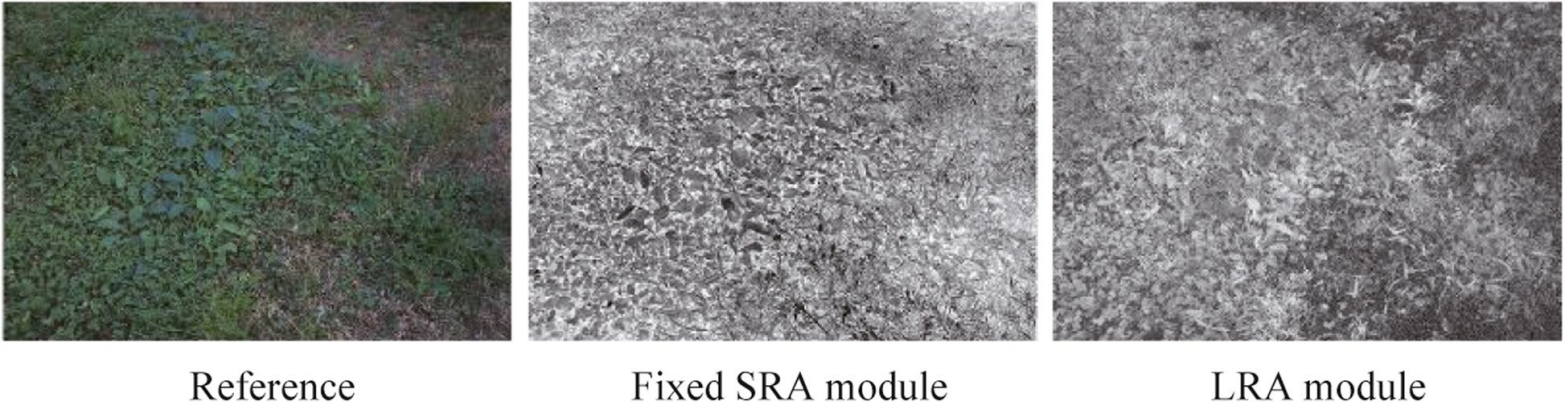
Attention maps generated with fixed SRA module and proposed LRA module. Both maps are normalized to [0, 1] for display

Subsequently, we compare the usefulness of illumination attention maps generated by different attention modules. B1 without any illumination attention module, B2 utilizes a self-regularized attention module with fixed coefficients, and proposed DFEN adopt a learnable regularized attention module. As depicted in Fig. 11, B1 is struggles to perceive the lighting conditions in various regions of the input image without the illumination attention module. This can result in overexposure of bright areas in the input image during enhancement. Therefore, in order to balance the enhancement effect of different regions, we introduce the SRA and LRA modules to extract the illumination attention map of the input images. However, the SRA module is hard to distinguish regions with similar brightness but different colors, which leads to color distortion in the vegetation part of the enhanced image. The LRA module uses a learnable convolution to generate illumination attention maps, making the boundaries of vegetation and dry grassland areas clearer. In addition, Fig. 12 displays the illumination attention maps generated with the learnable weights and fixed coefficients. It is obvious that the learnable approach can better distinguish different regions.

It should be noted that having a sufficient number of images with varied lighting conditions in the dataset is crucial for the efficiency of the LRA module. If the lighting conditions are too homogeneous, the LRA may fail and misrepresent the features of the enhanced images. For instance, excluding the images of normal illumination scenes from the dataset can significantly reduce the ability of the LRA to suppress overexposure.

#### Ablation experiments of training strategy

Finally, we evaluate the impact of different training strategies, the results of which are recorded in Table 5. C1–C4 only perform the loss constraint on the output image $I_{{{\text{Out}}}}$, C5–C7 add the constraint on the intermediate image $I_{{{\text{Mid}}}}$, and C8 adopts the same loss function configuration as the DFEN model but dose not adopt the proposed cosine training strategy. It shows that simultaneously adopt five losses during training helped to obtain better enhancement effect. When we remove the cosine training strategy, the enhancement effect gets worse. It may be caused by the inconsistent goals of the two training stages, making the training effect difficult to pass on, which is equivalent to shorten the number of valid training epochs. By establishing different loss constraints for the two stages output images and using the cosine training strategy to gradually adjust the loss weights of two stages during the training process, the two stages of the algorithm can focus on different task respectively, thus achieving better enhancement results.

In summary, the DBFF module, the LRA module and the cosine training strategy with five losses adopted by DFEN can effectively improve the enhancement performance, making the color more realistic, the detail clearer, and the enhance different regions more adaptable.

### Lighting conditions adaptability evaluation

In real-world applications, diverse lighting conditions pose a great challenge to low-light enhancement algorithms. In our Dark Grids dataset, we take images with various exposure duration in the same scene, which makes them have different lighting conditions. We use them to test the light condition adaptation of the DFEN and state-of-the-art methods.

**Fig. 13:**
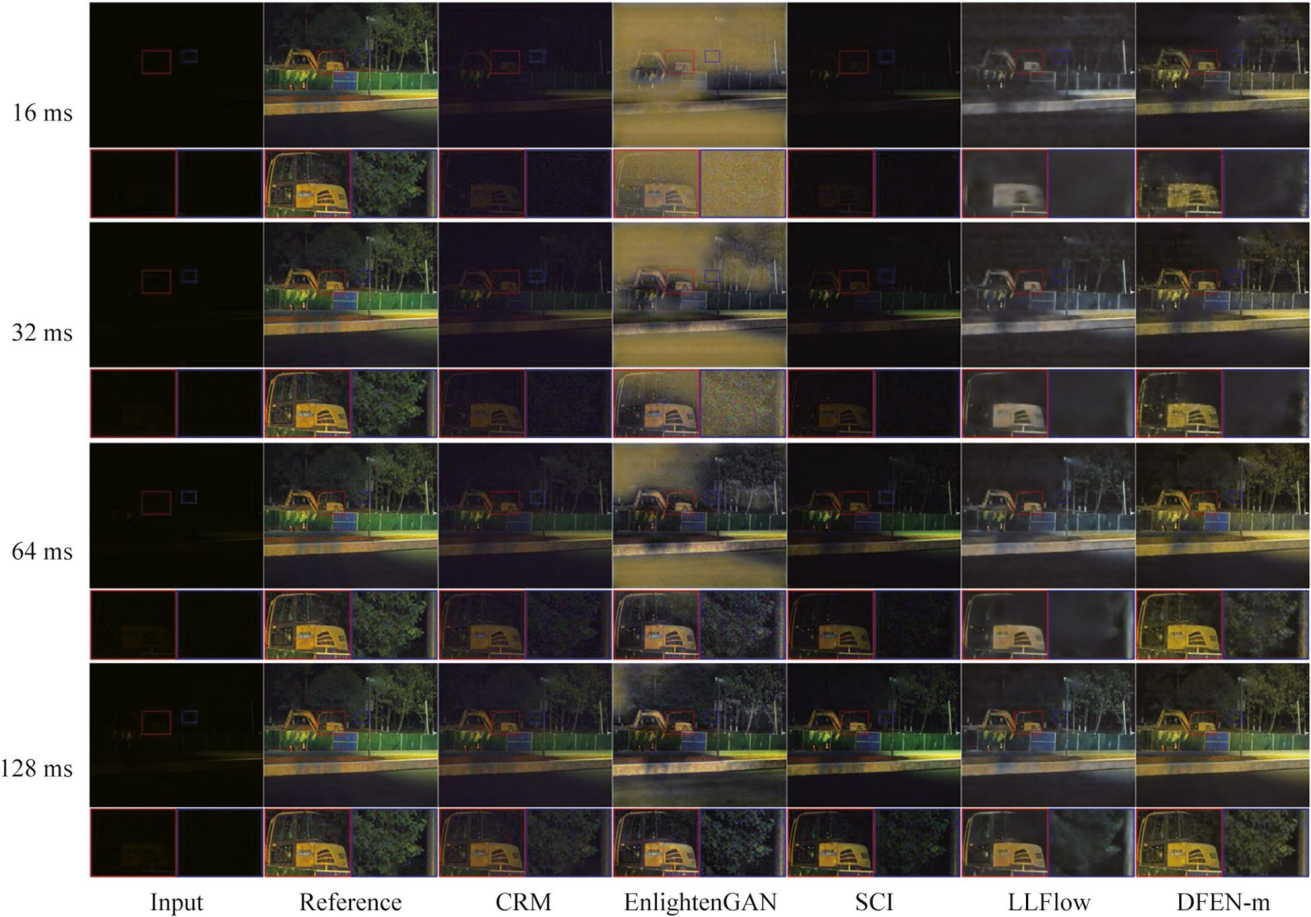
Visual comparisons of lighting conditions adaptation in the dark night scene

**Fig. 14:**
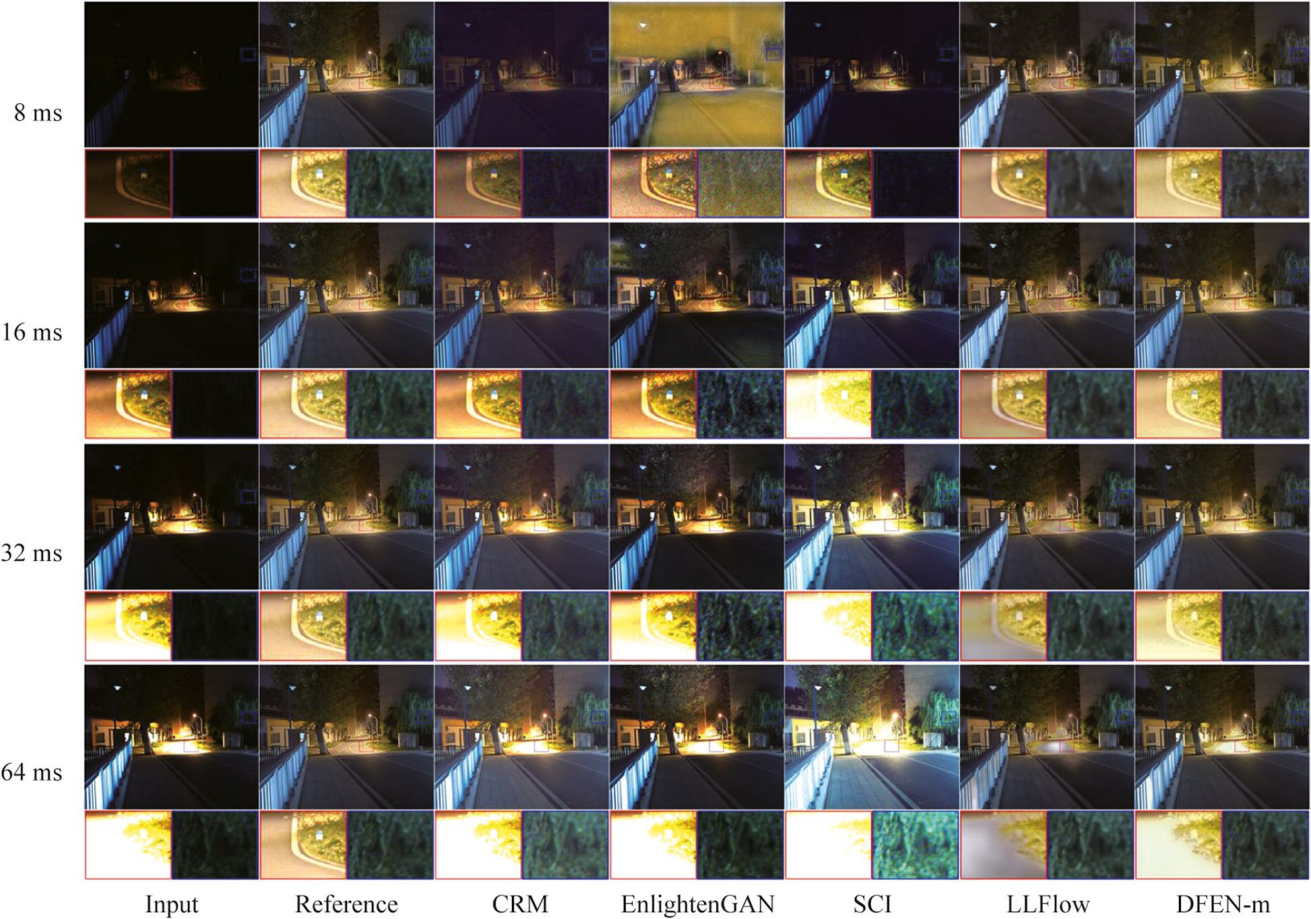
Visual comparisons of lighting conditions adaptation in the high dynamic range scene

As shown in Figs. 13 and 14, it can be seen that our algorithm has a more satisfactory adaptability to lighting conditions. Specifically, for the night dark scene shown in Fig. 13, DFEN can better recover the color and texture information of the low-light image when the exposure duration reaches 32 ms. However, the brightness of the results of CRM and SCI is low, the enhanced image of EnlightenGAN has serious artifacts, and LLFlow is difficult to recover the color information of the image. For the high dynamic range scene shown in Fig. 14, at 8 ms exposure time, only DFEN and LLFlow effectively enhance the detail information of the leaves in the shadows. And observe the pavement under the street lamp, when the exposure duration reaches 32 ms or more, CRM, EnlightenGAN and SCI all show more serious over-exposure phenomenon, and LLFlow produced white artifacts, while only DFEN could avoid the aggravation of over-exposure in the input image.

It can be seen that the exposure duration limits the ability of the camera to capture the environmental information in low-light scenes. Therefore, we need to extend the exposure time of the camera to obtain visually better enhanced images, but also to avoid the motion blur caused by long exposure time.

## Conclusion

To achieve rapid and premium enhancement of low-light images of power grid inspection scenes, we propose a two-stage end-to-end low-light enhancement algorithm DFEN. Compared with the one-stage method, DFEN decomposes the low-light enhancement task, making the learning target of each network more simplified. By employing the proposed cosine training strategy, it dynamically adjusts the loss function of the model. This allows the algorithm to focus on the learning of brightness adjustment and detail revision networks separately during different training epochs, thus achieving better enhancement results. In addition, we also adopt DBFF and LRA to further enhance the feature extraction and recovery ability of the model. Finally, we introduced a size control hyperparameter to adjust the number of channels in the U-Net. This allows our algorithm to flexibly balance the model size and enhancement effect based on practical application needs.

We also produce the Dark Grids dataset with various scenarios, and verify the effectiveness of the proposed method on several datasets including it. The results show that compared to state-of-the-art methods, the proposed DFEN can achieve better enhancement performance with the similar parameters, and has excellent scene adaptability. Among them, the lightest DFEN model reaches 11 FPS for image size of 1224×1024 in an RTX 3090 GPU.

We will continue to work on two aspects in the future. Firstly, the construction of paired datasets is complicated, so we are trying to introduce an unsupervised training strategy to get rid of the dependence on high-quality paired datasets. Second, the research and experiments of the algorithms are currently conducted on the server with an RTX 3090 GPU. We will complete the model deployment and inference acceleration on the edge computing platform, so that the model can be actually used on the grid inspection platform.

## Data Availability

The data that support the findings of this study are available from the corresponding author, upon reasonable request.
